# Lack of correlation between MYCN expression and the Warburg effect in neuroblastoma cell lines

**DOI:** 10.1186/1471-2407-8-259

**Published:** 2008-09-14

**Authors:** Danielle J Smith, Luke R Cossins, Irene Hatzinisiriou, Michelle Haber, Phillip Nagley

**Affiliations:** 1 Australian Research Council Centre of Excellence in Structural and Functional Microbial Genomics, Monash University, Wellington Road, CLAYTON, Victoria, 3800, Australia; 2 Department of Biochemistry and Molecular Biology, Monash University, Wellington Road, CLAYTON, Victoria, 3800, Australia; 3 Children's Cancer Institute Australia for Medical Research, High Street, RANDWICK, New South Wales, 2031, Australia

## Abstract

**Background:**

Many cancers preferentially meet their energy requirements through the glycolytic pathway rather than via the more efficient oxidative phosphorylation pathway. It is thought that this is an important adaptation in cancer malignancy. We investigated whether use of glycolysis for energy production even in the presence of oxygen (known as the Warburg effect) varied between neuroblastoma cell lines with or without *MYCN *amplification (a key indicator of poor disease outcome in neuroblastoma).

**Methods:**

We examined ATP and lactate production, oxygen consumption and mitochondrial energisation status for three neuroblastoma cell lines with varying degrees of *MYCN *amplification and MYCN expression.

**Results:**

We found no correlation between MYCN expression and the Warburg effect in the cell lines investigated.

**Conclusion:**

Our results suggest preferential use of glycolysis for energy production and MYCN expression may be independent markers of neuroblastoma malignancy *in vitro *if not *in vivo*.

## Background

Neuroblastoma is a common malignant disease of early childhood that exhibits a broad spectrum of clinical behaviour. As it is a disease of the sympaticoadrenal lineage of the neural crest, tumours can originate anywhere in the sympathetic nervous system[1]. Risk is stratified based on age (reduced risk accompanies detection prior to 18 months of age), histopathological features and *MYCN *amplification status[1]. *MYCN *is a member of the *MYC *family of oncogenes and is over-expressed preferentially in tumours of neuroectodermal origin, particularly neuroblastoma[2]. *MYCN *was the first amplified oncogene with clinical significance identified, and its amplification is highly correlated with advanced neuroblastoma disease stage, aggressive growth and poor prognosis[3].

The mechanism by which the MYCN transcription factor contributes to tumourigenesis remains unclear, although it has been shown to require gene amplification or protein stabilisation rather than mutation of the coding sequence[4]. In a transgenic mouse model of neuroblastoma in which human MYCN (hMYCN) was targeted to neural crest cells, tumours develop similar to human neuroblastoma in respect to their location (primary and metastatic), histology, syntenic chromosomal changes and common amplification of *hMYCN*[5-7]. Administration of MYCN antisense oligonucleotides to these mice inhibits gene expression (by blocking translation or splicing of RNA or by degrading target RNA[8]) and results in decreased tumour incidence, decreased tumour mass and increased morphological differentiation[9]. However, it appears *MYCN *is a conditionally favourable gene in neuroblastomas that do not have *MYCN *amplification[10,11] and the effect of *MYCN *expression in neuroblastomas from children of different ages or with disseminated disease may vary[11,12]. Thus, although the *MYCN *expression level itself is not a strong prognostic indicator, *MYCN *amplification and its attendant increase in MYCN protein remains one of the strongest indicators of the neuroblastoma malignant phenotype.

Neuroblastomas, like all tumours, must meet specific metabolic requirements to fuel their dysregulated growth and invasion into surrounding tissues. In most mammalian cells, glucose is catabolized to pyruvate that is further oxidized by mitochondrial oxidative phosphorylation to produce more than 30 ATP per glucose molecule. In the presence of oxygen, the catabolism of glucose to lactic acid and 2 ATP (glycolysis) is inhibited ('Pasteur effect'). However, up-regulation of glycolysis in the presence of oxygen ('Warburg effect'[13]) has been inferred in many cancers, including neuroblastomas[14], by the use of imaging technology to visualise the avid uptake of ^18^fluorodeoxyglucose. Indeed, up-regulated glycolytic capability and overall tumour aggressiveness is being recognised as a common trait of many cancers (for example, see recent review[15]). Certainly, a limited study of MYCN-inducible genes in a neuroblastoma cell line transfected with *MYCN *showed genes involved in glycolysis were up-regulated compared to the non-transfected parental neuroblastoma cell[16].

Here, we investigated whether there was a correlation between up-regulated glycolytic capabilities and the level of MYCN expression of three cell lines derived from patients with neuroblastoma who subsequently died of the disease. BE(2)-C cells were isolated from a two year old male who relapsed following intensive multiagent chemotherapy[17], have significant *MYCN *amplification[18], are highly tumorigenic in nude mice[19] and appear to represent the classic *MYCN*-amplified, highly aggressive neuroblastoma phenotype. SH-EP cells are a substrate-adherent sub-clone of the SK-N-SH cell line isolated from a bone marrow metastases of a four year old female[17,20]. SH-EP cells do not show *MYCN *amplification, have barely detectable levels of MYCN[2,21] and are completely non-tumorigenic in nude mice[22] thus representing the opposite end of the biological spectrum for neuroblastoma phenotype in comparison to BE(2)-C cells. NBL-S cells have an intermediate malignant phenotype, showing no amplification of *MYCN *but having a significantly prolonged MYCN half-life, and NBL-S cells are tumorigenic in nude mice[23]. We found here that in these three neuroblastoma cell lines elevated MYCN expression levels did not correlate with up-regulation of the Warburg effect or a concomitant reduction in cellular reliance on mitochondrial bioenergetic contribution.

## Methods

### Cell lines and culture

BE(2)-C and SH-EP cells were generously supplied by Dr. J. Biedler (Memorial Sloan-Kettering Cancer Centre, New York, NY). NBL-S cells were kindly supplied by Dr. S. Cohn (University of Chicago, Chicago, IL). The cell lines were maintained in Dulbecco's modified Eagle's medium (DMEM) supplemented with 10% (15% for NBL-S) heat-inactivated fetal calf serum, 2 mM L-glutamine and 10 mM HEPES.

### RT-PCR of MYCN

Real-time (RT) PCR analysis of *MYCN *gene expression, using *β*_2_-*microglobulin *as an internal control, was performed on aliquots of cDNA from each cell line corresponding to 50 ng of RNA as previously described[9].

### Western immunoblotting of MYCN

1 × 10^7 ^cells for each cell line were lysed and 30 μg of protein resolved by SDS-PAGE, transferred to nitrocellulose membrane and membranes stained for MYCN as previously described[11].

### ATP production

5 × 10^5 ^cells were seeded per well of six well tissue culture plates and incubated 48 hours. Media covering cells was replaced with fresh DMEM, DMEM plus 0.2 μM rotenone (Sigma), DMEM containing pyruvate but no glucose (PNG media) (JRH Bioscience) or PNG media plus rotenone for 1 hour prior to cell lysis on ice in 25 mM Tris-phosphate pH 7.8, 10% glycerol, 1% Triton-X100, 1 mg/ml BSA, 2 mM EDTA, 2 mM DTT. 100 μl of sample was added per well of black 96 well plates and 100 μl of D-Luciferin buffer (90 mM DTT, 20 mM Tricine, 8 mM MgCl, 0.13 mM EDTA, 1.4 mM D-Luciferin, 0.8 mM acetyl CoA) added per well. 100 μl of Luciferase buffer (90 mM DTT, 20 mM Tricine, 8 mM MgCl, 0.13 mM EDTA, 20 kU/ml D-Luciferase, 0.8 mM acetyl CoA) was injected and luminescence per well measured using a Fluostar Optima plate reader. A set of standards of known ATP concentration was assayed simultaneously, allowing ATP quantification for each sample.

### Protein content determination

Protein content in samples was measured using the Pierce Biotechnology (Rockford, IL) bicinchoninic acid (BCA) Protein Assay Kit as per manufacturers instructions.

### Oxygen consumption

Oxygen consumption was measured as previously described[24]. 1 × 10^6 ^neuroblastoma cells were incubated 48 h prior to collection by trypsinisation and resuspension in fresh, aerated Tris-based, Mg^2+^-, Ca^2+^-deficient (TD) buffer (137 mM NaCl, 5 mM KCl, 0.7 mM Na_2_HPO_4_, 25 mM Tris·HCl pH 7.4, at 25°C). 300 μl of each sample was loaded into a 37°C water-jacketed oxygraph chamber containing a small magnetic stirrer and connected to a circulating water bath at 37°C and a biological oxygen monitor (Strathkelvin Instruments, Scotland). Samples were read directly or after addition of 1 mM sodium azide for determination of azide-corrected oxygen consumption[25]. Oxygen consumption was recorded over time and normalised to mg of protein (determine by BCA assay) for each sample.

### Determination of mitochondrial energisation status

Cellular content of mitochondria and changes in mitochondrial membrane potential (Δψm) were determined as previously described[26]. 1 × 10^6 ^neuroblastoma cells were incubated 48 h prior to collection by trypsinisation and resuspension in DMEM plus 150 nM tetramethyl rhodamine methyl ester (TMRM; Invitrogen). Cells were incubated at 37°C for 20 min, washed in DMEM and incubated for a further 20 min in DMEM plus 100 nM MitoTracker Green (MTG; Invitrogen). Cells were washed and resuspended in PBS prior to analysis by flow cytometry (Becton Dickinson FACSCalibur with CellQuest software).

### Extracellular lactate levels

Extracellular lactate levels were measured as previously described[27]. 2 × 10^6 ^cells were seeded in 10 cm^2 ^tissue culture dishes and incubated for 48 hours. Culture media was replaced with DMEM plus HEPES with or without rotenone and incubated one hour. The media was removed, cell debris pelleted and 150 μl of each cell supernatant added per well of 96 well plate. 150 μl of glycine-hydrazine buffer (0.64 M glycine, 0.64 M hydrazine, 4.8 mM NAD^+^, 2 U/ml LDH) was injected per well and reduction of NAD^+ ^by lactate dehydrogenase was monitored spectrophotometrically at 340 nm using a Fluostar Optima plate reader. A set of standards of known lactate concentration was assayed simultaneously, allowing quantification of lactate for each sample.

## Results

### Neuroblastoma cell lines BE(2)-C, NBL-S and SH-EP have different levels of MYCN expression

**Figure 1:**
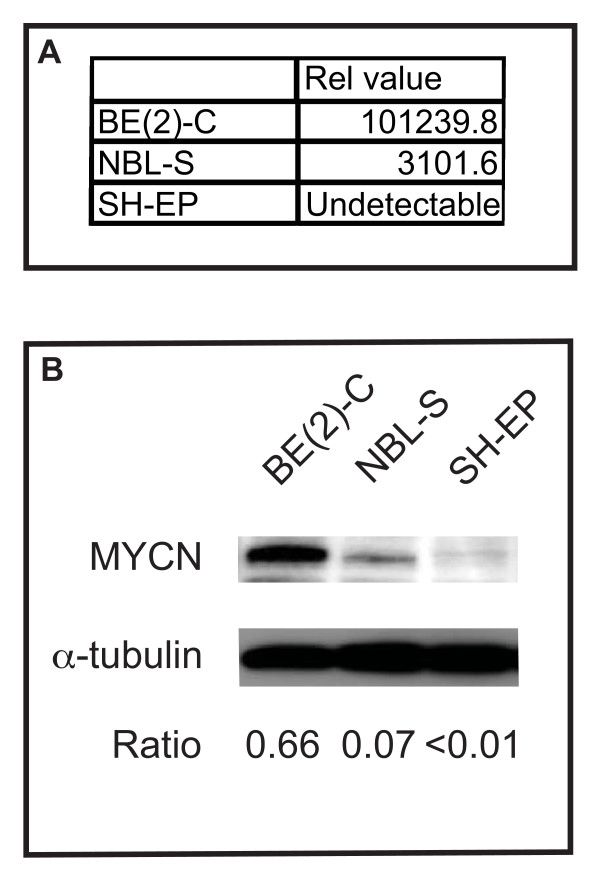
**Constitutive expression of MYCN in BE(2)-C, NBL-S and SH-EP neuroblastoma cell lines**. (A) Levels of *MYCN *mRNA in neuroblastoma cell lines. mRNA from neuroblastoma cell lines was examined using real-time PCR for *MYCN *levels. *β*_2_-*microglobulin *was used as a control gene. Relative (rel) value represents the abundance of *MYCN *transcript relative to control gene. (B) BE(2)-C, NBL-S and SH-EP cell lines express different levels of MYCN. Lysates from neuroblastoma cell lines were analysed by Western immunoblotting using an anti-MYCN antibody. α-tubulin was used as a protein loading control. Ratio represents intensity of MYCN relative to α-tubulin.

*MYCN *is an oncogene that is critical in the pathogenesis of neuroblastoma. *MYCN *mRNA and protein levels for BE(2)-C, NBL-S and SH-EP neuroblastoma cell lines were investigated using RT-PCR and Western immunoblotting (Figure 1). The *MYCN*-amplified BE(2)-C cell line had the highest *MYCN *mRNA content of the neuroblastoma cell lines investigated, being approximately 30 times greater than the next highest, NBL-S (Figure 1A). BE(2)-C cells also expressed more MYCN than the other cell lines, having a MYCN:α-tubulin protein ratio of 0.66 compared with a ratio of just 0.07 for NBL-S and < 0.01 for SH-EP (Figure 1B). The low level (by RT-PCR) of *MYCN *in SH-EP cells appears to account for the minimal MYCN expression in this cell line (which is not amplified for the *MYCN *gene).

### ATP production by glycolysis and oxidative phosphorylation in neuroblastoma cell lines

**Figure 2:**
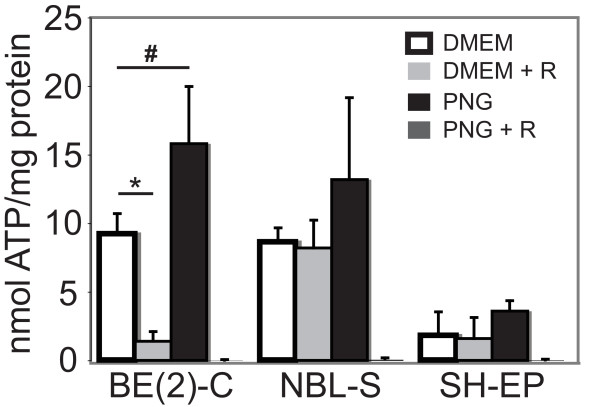
**ATP synthesis in neuroblastoma cells utilising glycolysis or oxidative phosphorylation**. The ATP content per mg of total cell protein under different energy substrate restriction conditions was determined spectrophotometrically using a luciferase-based reporter assay. Cells were cultured in □ normal culture medium (DMEM);  conditions inhibiting oxidative phosphorylation (DMEM plus rotenone); ■ conditions inhibiting glycolysis (DMEM containing pyruvate but no glucose (PNG media));  conditions inhibiting both oxidative phosphorylation and glycolysis (PNG plus rotenone). BE(2)-C cells produced significantly different (*p < 0.01, #*p *< 0.05) levels of ATP under conditions inhibiting oxidative phosphorylation or glycolysis compared to that produced in normal culture media.

Unregulated growth and survival is an energy-demanding process of tumourigenesis, yet malignant cancers often down-regulate oxidative phosphorylation in favour of less efficient glycolytic energy production. We investigated the energy production capacity of the three neuroblastoma cell lines to determine if they displayed elevated glycolytic capacity. Neuroblastoma cells were incubated under conditions inhibiting either oxidative phosphorylation (rotenone treatment) or glycolysis (glucose-free, pyruvate-supplemented media)[28] for one hour prior to measurement of ATP levels (Figure 2). SH-EP and NBL-S cells appeared as capable of producing ATP in experimental conditions restricting oxidative phosphorylation or glycolysis as they were under normal culture conditions (where both oxidative phosphorylation and glycolysis can contribute to ATP production). BE(2)-C cells, however, showed a marked reduction in their ability to produce ATP (from that seen under normal culture conditions) when oxidative phosphorylation was inhibited, although they showed enhanced capacity to generate ATP when supplied with excess pyruvate as an energy substrate in the presence of oxygen. Thus the *MYCN*-amplified BE(2)-C cells appeared reliant on oxidative phosphorylation to meet their energy requirements.

### Lactate production by neuroblastoma cell lines as a measure of glycolytic capability

**Figure 3:**
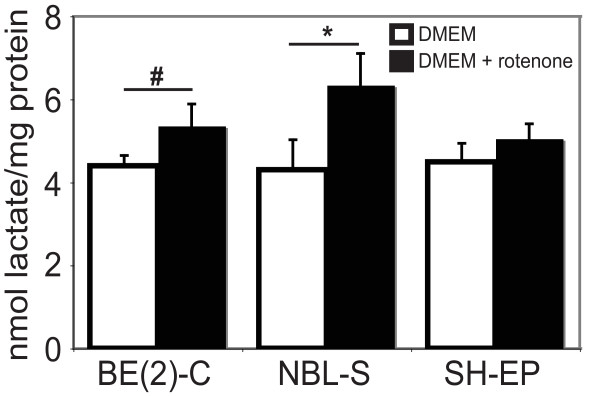
**Neuroblastoma cell lines produced lactate in the presence of the oxidative phosphorylation inhibitor, rotenone**. Culture media from neuroblastoma cell lines incubated ■ with or □ without rotenone to inhibit oxidative phosphorylation was assayed for extracellular lactate as a measure of glycolysis. BE(2)-C and NBL-S cell lines produced significantly more (*p < 0.01, #*p *< 0.05) lactate per mg of total cellular protein when cultured in the presence of rotenone.

Pyruvate generated from glucose catabolism can be reduced to lactic acid in the absence of oxygen (glycolysis) or further metabolised by the mitochondria in the presence of oxygen. BE(2)-C, NBL-S and SH-EP cell cultures were examined for extracellular lactate levels as an indicator of glycolysis (Figure 3). Measurements were taken from cells incubated under normal culture conditions or cultured with rotenone to inhibit oxidative phosphorylation. All cell lines showed an increase (above untreated levels) in lactate after one hour rotenone treatment, with the biggest increase observed for NBL-S cell lines where lactate levels increased by over 30%. These results support our findings that while the three neuroblastoma cells could use glycolysis when oxidative phosphorylation was inhibited, they did so with varying degrees of efficiency.

### Oxygen consumption as a measure of oxidative energy production capacity correlated with MYCN expression level in neuroblastoma cell lines

**Figure 4:**
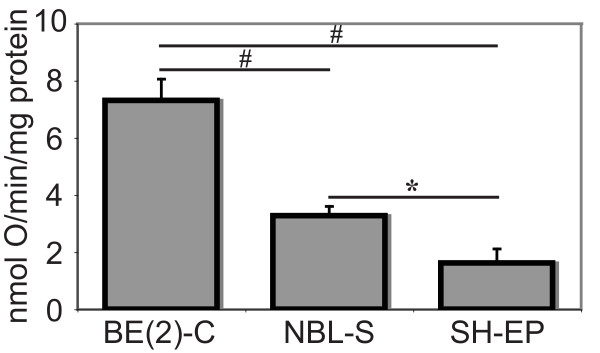
**BE(2)-C cells consumed more oxygen than NBL-S or SH-EP cell lines**. Oxygen consumption, as a measure of total oxidative ATP production, was measured using polarography and the azide-corrected oxygen consumption of each cell line per mg of protein was determined. Levels of oxygen consumption were statistically different (*p < 0.05, #*p *< 0.005) between the various cell lines.

The final stages of energy production via oxidative phosphorylation involves the direct consumption of molecular oxygen[29]. Oxygen consumption (as a measure of total oxidative ATP production) was measured for the three neuroblastoma cell lines using polarography and resultant data were expressed per milligram of protein for each cell line (Figure 4). Results were adjusted for the oxygen consumption specifically inhibited by sodium azide (i.e. mitochondrial). Of the neuroblastoma cell lines investigated, BE(2)-C cells had the highest oxygen consumption rate, followed by NBL-S. SH-EP cells consumed the least amount of oxygen per minute per milligram of protein, being significantly lower than NBL-S (*p *< 0.05) and BE(2)-C (*p *< 0.005). These results suggest BE(2)-C cells produced the majority of their energy via the oxygen-dependent, complete catabolism of glucose whereas SH-EP cells, and to a lesser extent NBL-S cells, had a lower dependency on oxygen when meeting their energy requirements.

### Malignant phenotype of neuroblastoma is associated with elevated mitochondrial energisation status

**Figure 5:**
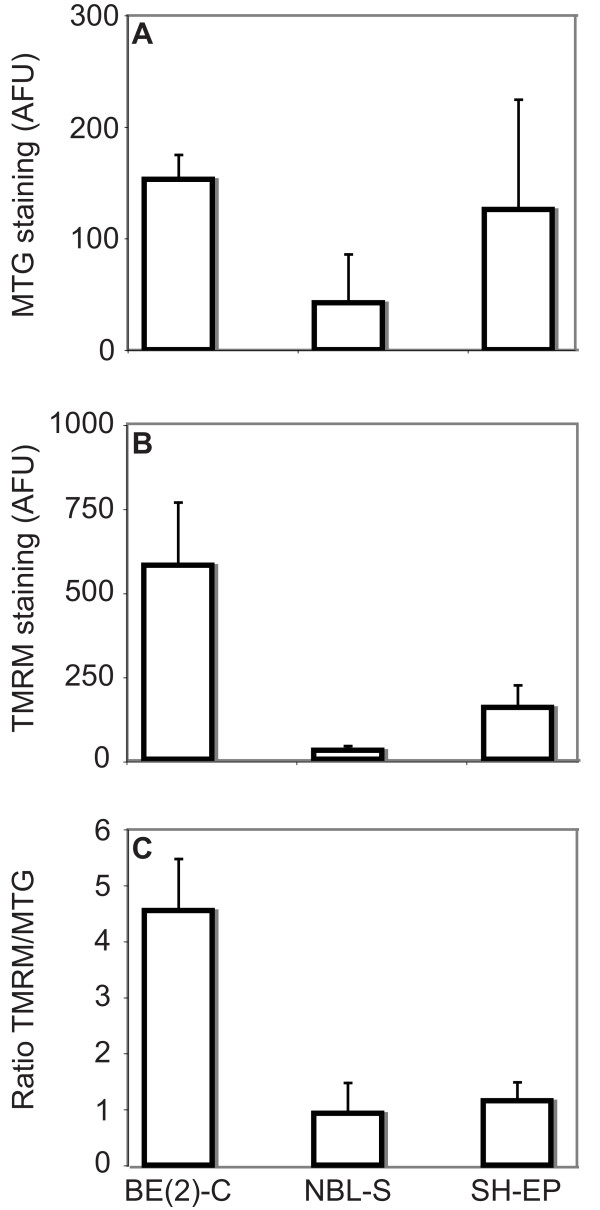
**Mitochondrial content and functionality of neuroblastoma cell lines**. Neuroblastoma cell lines were examined by flow cytometry for (A) MTG (as a measure of mitochondrial content) and (B) TMRM uptake (as a measure of mitochondrial membrane potential). (C) The ratio of TMRM and MTG staining represents the mitochondrial capability of each cell line. Cellular uptake of dyes is shown in arbitrary fluorescent units (AFU).

Studies in tumour cells have demonstrated a link between the use of glycolysis for energy production and attenuated mitochondrial bioenergetic capabilities[30,31]. This attenuation most likely occurs as a result of a decrease in the number of mitochondria per cell or reduction in the activity of mitochondrial respiratory complexes and ATP synthase[32]. Using flow cytometry[33], we examined the three neuroblastoma cell lines for mitochondrial membrane potential and mitochondrial mass per cell, using TMRM and MTG staining, respectively. The uptake of TMRM by mitochondria occurs in a membrane potential (Δψm)-dependent manner and is a measure of mitochondrial respiratory capacity. Mitochondrial abundance was measured by staining cellular mitochondrial content with MTG (signal intensity is independent of membrane potential). The ratio of mitochondrial Δψm to organelle mass per cell was used to determine the overall mitochondrial energisation status of the cell lines. Mitochondrial energisation differed between the three cell lines (Figure 5). SH-EP cells had relatively high mitochondrial abundance but low Δψm. NBL-S cells had a low number of mitochondria per cell and NBL-S mitochondria had a relatively low Δψm. BE(2)-C cells had a similar abundance of mitochondria to SH-EP cells but BE(2)-C mitochondria had significantly higher Δψm, correlating with the higher oxidative phosphorylation capacity of BE(2)-C cells (Figure 2). Thus, BE(2)-C cells had the greatest mitochondrial energisation capacity with SH-EP and NBL-S cell lines displaying a lower, but similar, capacity. This may leave NBL-S and SH-EP cells more reliant on an alternative to oxidative phosphorylation for their energy needs.

## Discussion

Otto Warburg first posited that a critical notion for the understanding of tumourigenesis is the increased preference for glycolysis in the presence of oxygen which he observed in cancer cells[13]. For many decades since then other researchers have tried to determine why this is so. Studies applying fluorodeoxyglucose positron emission tomography (FDG-PET) have identified increased glucose uptake, critical in ensuring adequate generation of fuel to meet cellular energy requirements via inefficient glycolysis, as a hallmark of metastatic cancers in humans. This phenotype, namely elevated glucose uptake, irrespective of oxygen availability, correlates with the aggressiveness of non-small cell lung carcinoma[34], lymphoma[35], glioma[36] and gastrointestinal[37] tumours. One may infer from the commonality of the phenomenon that such a phenotype confers a significant competitive advantage in carcinogenesis. Hypotheses for the preferential use of glycolysis regardless of oxygen availability by cancer cells include: ensuring ATP production in environments with limited oxygen (such as that occurring when tumour growth outpaces vascularity[38]) to fuel cell functions such as proliferation; production of reducing equivalents to mitigate reactive oxygen species (ROS) stress (ROS is produced by inefficiently respiring mitochondria)[39]; or generation of a toxic environment[40] which selects for cells with abrogated death signalling. We have examined here three neuroblastoma cell lines (derived from patients who ultimately died from the disease) for their glycolytic capacity, in order to determine whether increased aerobic glycolysis (Warburg effect) correlated with expression levels of MYCN, a factor implicated in the pathogenesis of neuroblastoma.

BE(2)-C cells expressing high levels of MYCN appeared to satisfy most of their energy requirements using oxidative phosphorylation; they consumed the greatest amount of oxygen and had the highest mitochondrial energisation capability. While the consumption of oxygen by BE(2)-C cells may consume oxygen to fuel ROS production[41] rather than ATP generation, we believe our data suggests that highly malignant BE(2)-C cells do not have a glycolytic energy production capacity sufficient to meet their requirements. Certainly, BE(2)-C cells showed an 85% decrease in ATP generation and only moderate increases in lactate production when oxidative phosphorylation was inhibited. NBL-S cells, by comparison, with a lower (but still elevated) level of MYCN expression compared to BE(2)-C, appeared to have up-regulated glycolytic capabilities, possessing the ability to maintain ATP production capacity and generate significant levels of lactate when oxidative phosphorylation was inhibited. SH-EP cells, without *MYCN *amplification and expressing no detectable MYCN, showed the lowest energy production capability and had no apparent preference for using either oxidative phosphorylation or glycolysis to meet their energy requirements. The reduction in use of oxidative phosphorylation by NBL-S and SH-EP cells may reflect their reduced reliance on it as the only pathway for energy generation.

Other groups have transfected cell lines with oncogenes other than *MYCN *(*Ras *or *Akt*) and have found that increasing transformation in these cell lines correlates not only with increasing oncogene expression but also aerobic glycolysis[42,43]. And transfection of SH-EP neuroblastoma cell lines with *MYCN *has also been shown to induce the up-regulation of several genes involved in glycolysis[16]. While our results may appear different than previously published finding, we believe potential discrepancies are due to the methodology of the studies. The other studies created situations where the only change between cells being examined were those experimentally induced. We, on the other hand, have examined cells derived from neuroblastomas that evolved into malignant tumours *in vivo *and presumably have many other adaptations to their environment than those simply regulating energy production. Indeed, when Boon et. al. analysed expression of genes involved in glycolysis (shown previously to be upregulated in SH-EP cell lines overexpressing exogenous MYCN) in two other neuroblastoma-derived cells lines (one with *MYCN *amplification and one with a single MYCN copy) there was almost no induction of glycolytic genes[16]. Thus, while the upregulation of glycolytic enzymes may be a factor in neuroblastoma malignancy, it may be independent of MYCN expression.

## Conclusion

It appeared that there is no correlation between MYCN expression and glycolytic adaptation in the neuroblastoma cell lines investigated. While we acknowledge this lack of correlation may not reflect *in vivo *bioenergetic capabilities of neuroblastoma, we propose that MYCN expression and an upregulated Warburg effect may be independent markers of neuroblastoma malignancy. Certainly, our results suggest PET technology to image elevated glucose uptake (compared with normal surrounding tissue) and treatments based on inhibiting tumour glycolysis may be relevant in only a sub-set of neuroblastomas[44].

## Competing interests

The authors declare that they have no competing interests.

## Authors' contributions

LC performed the experiments under DS guidance, with the exception of the expression of MYCN that was performed at CCIA under MH guidance. DS and PN designed the experiments with input from LC and IH. DS wrote the manuscript with input from LC and MH. PN and MH conceived of the study. All authors read and approved the manuscript.

## Pre-publication history

The pre-publication history for this paper can be accessed here:


